# Hepcidin and ferritin levels in restless legs syndrome: a case–control study

**DOI:** 10.1038/s41598-020-68851-0

**Published:** 2020-07-17

**Authors:** Sofiene Chenini, Constance Delaby, Anna-Laura Rassu, Lucie Barateau, Jérôme Vialaret, Christophe Hirtz, Anne Marie Dupuy, Sylvain Lehmann, Isabelle Jaussent, Yves Dauvilliers

**Affiliations:** 1 Sleep-Wake Disorders Unit, Department of Neurology, Gui-de-Chauliac Hospital, CHU Montpellier, Montpellier University, Montpellier, France; 2 National Reference Network for Narcolepsy, CHU Montpellier, Montpellier, France; 3 INSERM U1061, Neuropsychiatry: Epidemiological and Clinical Research, University Montpellier, 34295 Montpellier, France; 4 Laboratory of Biochemistry-Clinical Proteomic, CHU Montpellier, Montpellier University, INSERM U1040, Montpellier, France; 5 Department of Biochemistry, Montpellier University Hospital, Montpellier, France

**Keywords:** Neurological disorders, Neurology

## Abstract

The association between restless legs syndrome (RLS) and iron homeostasis remains unclear. We compared serum hepcidin and ferritin levels in patients with RLS and controls, and assessed their relationships with RLS phenotype, drug intake, and history of augmentation syndrome. 102 drug-free RLS patients (age 58.9 [24.5–77.2], 63 females) and 73 controls (age 56.8 [23.46–76.6], 45 females) underwent a polysomnography recording. Hepcidin levels were quantified by ELISA. 34 RLS patients had a second assessment after starting dopaminergic drugs. Ferritin level was low (< 50 µg/l) in 14.7% of patients and 25% of controls, with no between-group differences in the mean values. Hepcidin levels were higher in patients even after adjustment for confounding factors, and excluding participants with low ferritin levels. Ferritin and hepcidin levels were comparable before and after treatment, and between patients with (n = 17) and without history of augmentation. Ferritin and hepcidin levels correlated with age, body mass index, and periodic leg movements. Higher hepcidin levels were associated with older age, older age at RLS onset, less daytime sleepiness and familial RLS. In conclusion, serum hepcidin levels but not ferritin were higher in RLS patients regardless of treatment and history of augmentation. Serum hepcidin may be a more relevant biomarker of RLS than ferritin.

Restless legs syndrome (RLS) is a frequent sensorimotor disorder that often impairs sleep and quality of life^1, 2^. RLS is associated with periodic leg movements in sleep (PLMS) in 80% of cases^3^. Genetic burden, iron deficiency and dopamine dysregulation play a major role in the pathogenesis of RLS and PLMS^4–6^. Several neuropathological, biological, and brain-imaging studies have found iron deficiency in the brain and cerebrospinal fluid of patients with RLS^7^. However, the cause of such deficiency is still unclear.

Serum ferritin level is considered as a good biomarker of iron stores^8^. Although iron deficiency has been associated with RLS pathogenesis, low serum ferritin has only been reported in 10–20% of adults with RLS^7^. Moreover, recent large clinical and population-based studies did not confirm the association between RLS and serum ferritin levels^9–13^. One isolated study showed that low serum ferritin level is a potential biomarker for RLS augmentation^14^, one of the most severe complications of this syndrome. Augmentation is a treatment-induced paradoxical worsening of RLS symptoms caused by long-term high dose dopaminergic therapy, and remains the major challenge of RLS management^14–16^.

Hepcidin is the main regulator of iron homeostasis^17^, by modulating the internalization and degradation of the iron-exporter ferroportin, and thus inhibiting iron absorption from the gut and iron release from storage sites. Consequently, hepcidin has emerged as a strong biomarker for the diagnosis and monitoring of a large spectrum of iron-related disorders^18^. As a dynamic iron regulator, high hepcidin expression will decrease plasma iron levels, whereas low hepcidin expression will increase iron concentration. We recently reported higher serum hepcidin levels in drug-free patients with primary RLS and normal ferritin levels compared with controls. Hepcidin, but not ferritin, was associated with RLS severity and PLMS^9^. In this previous study, we quantified bioactive hepcidin-25 using a liquid chromatography-tandem mass spectrometry (LC–MS/MS)-based quantitative method, which is the reference method for peptide quantification. However, it is a complex and time-consuming approach and this limits its application in the clinical routine. Other techniques have been developed for hepcidin detection and quantification in human blood samples, such as radio-immunoassays and enzyme-linked immunosorbent assays (ELISA). Recent comparative studies showed good analytical performances of these methods in terms of reproducibility, linearity, and mutual correlations^19^. Nowadays, the commercial ELISA assay is a reliable tool for measuring serum hepcidin in humans^19,20^. To our knowledge, serum hepcidin levels have never been measured in RLS patients using the easy-to-use ELISA assay, and in the context of iron deficiency, dopaminergic drug intake and recent history of augmentation.

The objectives of the present study were (1) to compare serum hepcidin and ferritin levels in patients with RLS and controls using the ELISA assay for hepcidin, (2) to assess the relationships between hepcidin and ferritin levels in patients with RLS as function of iron levels, RLS severity, polysomnographic characteristics, dopaminergic drug intake, and recent history of augmentation syndrome, and (3) to compare two methods of serum hepcidin measurements (ELISA and LC–MS/MS) in a subgroup of RLS patients and controls.

## Results

### Clinical and polysomnographic characteristics of drug-free patients with RLS and controls

In drug-free patients with RLS, the median ages at RLS onset and RLS diagnosis were 43 years (6–65 years) and 49 years (15–73 years), respectively. Family history of RLS was reported by 41.3% of patients. All patients had a severity score higher than 15, and 83% had severe RLS (score > 20). Among these patients, only 40 (39.2%) were drug naive (i.e., never exposed to RLS medications), whereas the others stopped the medications for RLS or any other medication known to influence sleep or movement at least 2 weeks prior to the clinical and sleep assessment for research purposes, or for switching to another treatment after testing.

Body mass index (BMI) as well as the ESS, ISI and BDI II scores were higher in patients than controls, indicating higher levels of sleepiness, insomnia and depressive symptoms (Table 1). As expected, PLMS, PLM in Wakefulness (PLMW), micro-arousal index, and wake after sleep onset (WASO) were higher, and total sleep time lower in patients with RLS than in controls.

**Table 1:** Demographic, clinical and polysomnographic characteristics of drug-free patients with restless legs syndrome (RLS) and controls.

Variable	Controls N = 73		Drug-free patients with RLS N = 102		p value
	n	%	n	%	
**Demographic characteristics**					
Sex					
Male	28	38.36	39	38.24	0.99
Female	45	61.64	63	61.76	
Age, years^a^	56.85 (23.46; 76.65)		58.91 (24.50; 77.22)		0.20
BMI, kg/m^2^	23.41 (18.26; 34.53)		25.58 (18.34; 40.40)		0.001
BMI, kg/m^2^					
< 25	52	71.23	47	46.08	0.004
25–30	19	26.03	44	43.14	
> 30	2	2.74	11	10.78	
**Clinical characteristics**					
Hypertension, Yes	10	13.70	16	15.84	0.70
Hypercholesterolemia, Yes	7	9.59	14	14.29	0.36
Diabetes mellitus, Yes	2	2.74	4	3.96	0.67
ESS total score^a^	6.00 (0.00; 15.00)		10.50 (0.00; 23.00)		< 0.0001
ESS total score, > 10	13	17.81	49	50.00	< 0.0001
ISI total score^a^	5.00 (0.00; 19.00)		18.00 (4.00; 28.00)		< 0.0001
ISI total score					
< 8	45	64.29	4	4.44	< 0.0001
[8–15]	20	28.57	19	21.11	
≥ 22	5	7.14	67	74.44	
BDI II total score^a^	5.00 (0.00; 25.00)		12.00 (1.00; 45.00)		< 0.0001
BDI II total score					
≤ 13	64	91.43	50	54.95	< 0.0001
14–19	3	4.29	15	16.48	
20–63	3	4.29	26	28.57	
**Polysomnography measurements**					
Total sleep time, min^a^	364.00 (146.00; 525.00)		321.50 (53.00; 480.00)		0.0005
Sleep efficiency, %^a^	75.00 (28.37; 94.12)		71.25 (11.00; 6,300.00)		0.58
Wake time after sleep onset, min^a^	82.00 (17.00; 263.00)		102.00 (13.00; 404.00)		0.01
Sleep latency, min^a^	16.00 (0.00; 146.00)		16.50 (0.00; 300.00)		0.41
REM sleep latency, min^a^	93.00 (20.00; 207.00)		91.50 (12.00; 453.00)		0.22
N1, %^a^	6.48 (1.30; 35.79)		7.00 (1.88; 29.70)		0.31
N2, %^a^	54.79 (33.56; 74.68)		54.81 (30.69; 81.38)		0.64
N3, %^a^	20.27 (3.17; 42.12)		17.42 (0.00; 45.40)		0.11
REM sleep, %^a^	16.58 (2.04; 29.17)		20.13 (0.00; 43.21)		0.06
Microarousal index, /h^a^	14.00 (1.00; 46.00)		22.50 (0.00; 181.00)		0.0001
PLM during sleep index, /h^a^	1.15 (0.00; 71.06)		38.60 (0.00; 234.32)		< 0.0001
PLM during sleep index, /h, ≥ 15	11	15.07	67	65.69	< 0.0001
PLM during wake index, /h^a^	7.56 (0.00; 108.29)		34.51 (0.00; 250.00)		< 0.0001
Apnea–hypopnea index, /h^a^	3.77 (0.00; 40.81)		6.05 (0.00; 55.84)		0.16
Apnea–hypopnea index, /h ≥ 15	10	13.70	22	21.57	0.19
SaO_2_^a^	95.00 (91.00; 98.00)		95.00 (91.00; 98.00)		0.90

*BMI* body mass index (weight in kg divided by the square of the body height in meters), *BDI* Beck Depression Inventory, *ESS* Epworth Severity Scale, *ISI* Insomnia Severity Index, *PLM* periodic legs movements, *REM* rapid eye movement, *SaO*_*2*_ oxygen saturation.

^a^Quantitative variables are expressed as median (minimum value; maximum value).

### Ferritin and hepcidin quantification in drug-free patients with RLS and controls

Compared with controls, serum ferritin levels were slightly higher in the RLS group in unadjusted associations, but not after adjustment for BMI (Table 2). Similar results were obtained when ferritin levels were divided into tertiles. Ferritin levels were low (< 50 µg/L) in 15 (14.71%) patients with RLS and in 18 (25%) controls, with no between-group difference. None of participants were taking iron replacement therapy for at least 2 years.

**Table 2:** Serum hepcidin and ferritin quantification in drug-free patients with restless legs syndrome (RLS) and controls.

Iron measurements	Controls		Drug-free patients with RLS		Model 0^e^		Model 1^f^	
	N = 73		N = 102					
	n	%	n	%	OR [95% CI]	p-value	OR [95% CI]	p-value
Serum ferritin, µg/L^a,b^	118.00 (13.00; 567.00)		151.50 (7.00; 536.00)		1.14 [0.99; 1.30]	0.05	1.08 [0.94; 1.24]	0.28
**Serum ferritin, µg/L**								
< 50	18	24.66	15	14.71	1	0.10	1	0.21
≥ 50	55	75.34	87	85.29	1.90 [0.88; 4.07]		1.66 [0.75; 3.67]	
**Serum ferritin, µg/L**^**c**^								
< 94	30	41.10	26	25.49	1	0.07	1	0.23
[94–187]	24	32.88	36	35.29	1.73 [0.83; 3.62]		1.73 [0.81; 3.69]	
≥ 187	19	26.03	40	39.22	2.43 [1.14; 5.18]		1.85 [0.84; 4.10]	
Serum hepcidin, µg/L^a,d^	11.89 (0.00; 70.51)		18.36 (0.00; 77.17)		1.47 [1.13; 1.90]	0.004	1.40 [1.08; 1.81]	0.01
**Serum hepcidin, µg/L**^**c**^								
< 9.97	32	43.84	26	25.49	1	0.01	1	0.04
[9.97–20.99]	25	34.25	33	32.35	1.62 [0.78; 3.38]		1.29 [0.60; 2.78]	
≥ 20.99	16	21.92	43	42.16	3.31 [1.53; 7.16]		2.72 [1.22; 6.04]	

^a^Quantitative variables are expressed as median (minimum value; maximum value).

^b^OR for a 50-unit increase.

^c^Categorized into tertiles.

^d^OR for a 10-unit increase.

^e^Model 0: Crude association.

^f^Model 1: Model adjusted for BMI.

Conversely, hepcidin levels were significantly higher in patients than in controls, even after adjustment for BMI (median [ranges] 18.36 µg/L [0.00–77.17] vs 11.89 [0.00; 70.51], p < 0.01) (Table 2). Five patients with RLS and four controls had undetectable hepcidin levels (i.e. below the detection limit^26^). When hepcidin levels were divided into tertiles, we found that the number of patients in each tertile progressively increased in contrast to controls. ROC analyses were performed to determine the cutoff values for the hepcidin levels with the highest sensitivity and specificity to discriminate patients with RLS from controls. The area under the curve values was 0.62 (95% CI [0.55–0.71]) for hepcidin level. The best cut off hepcidin value to discriminate patients with RLS from controls was 18.1 µg/L, with a sensitivity of 52% and a specificity of 75%, and with significant between-group differences (p = 0.0004).

A sensitivity analysis of the participants with normal ferritin levels (> 50 µg/L; (87 patients and 55 controls) confirmed that hepcidin levels were higher in patients with RLS than in controls (median [ranges] 20.65 [0–77.17] vs 14.62 [0–70.51], p = 0.02), even after adjustment for BMI. Another sensitivity analysis after exclusion of participants included in a previous study in which hepcidin was measured by LC–MS/MS assay (42 patients with RLS and 33 controls) confirmed that hepcidin level was higher in patients with RLS than in controls (60 vs 40 subjects; median [ranges] 11.20 [0–77.17] vs 8.15 [0–70.51], p = 0.03), without significant difference in ferritin levels, even after controlling for BMI.

Ferritin level correlated with hepcidin level in the whole sample, in patients with RLS, and in controls (r = 0.57, 0.57, 0.54, p < 0.0001, respectively). In the whole sample, both ferritin and hepcidin levels slightly correlated with age (r = 0.25; r = 0.27, p < 0.001 respectively), BMI (r = 0.28; r = 0.20, p < 0.01 respectively), and PLMS (r = 0.19; r = 0.18, p < 0.015 respectively).

### Determinants of high serum hepcidin levels in drug-free patients with RLS

To identify factors that could be associated with high hepcidin levels in RLS, patients with RLS were divided in two groups based on the highest serum hepcidin tertile (*≥ *20.99 ng/mL and < 20.99 ng/mL). High hepcidin level (*≥ *20.99 ng/mL) was associated with older age, older age at RLS onset, older age at daily RLS onset, positive family history of RLS, lower daytime sleepiness (ESS score), lower total sleep time, and higher WASO, REM sleep latency, apnea–hypopnea index (AHI) and PLMW. RLS was very severe (score > 30) in 38.1% of patients with high hepcidin level and in 26.8% of patients with low hepcidin level (< 20.99 ng/mL). RLS was moderate in 40.5% of patients with high hepcidin level and in 64.3% of patients with low hepcidin level, suggesting a trend towards a non-linear relationship (U-shaped curve) between hepcidin level and RLS severity (p = 0.05) (Table 3). After adjustment for age, the associations between hepcidin levels, daytime sleepiness, family history of RLS, total sleep time, and WASO remained significant; however given the multiple testing performed these results should be taken with caution.

**Table 3:** Demographic, clinical and polysomnographic determinants of high hepcidin levels in patients with restless legs syndrome (RLS).

Variable	Hepcidin (µg/L)				Model 0	Model 1
	< 20.99 N = 59		≥ 20.99 N = 43			
	n	%	n	%	p-value	p-value
**Demographic characteristics**						
Sex, Female	37	62.71	26	60.47	0.82	0.62
Age, years^a^	53.15 (24.50; 74.95)		64.11 (37.59; 77.22)		0.002	
BMI, kg/m^2^	25.59 (18.34; 37.45)		25.56 (18.75; 40.40)		0.51	0.82
Hypertension, Yes	7	12.07	9	20.93	0.23	0.47
Hypercholesterolemia, Yes	6	10.71	8	19.05	0.24	0.63
Diabetes mellitus, Yes	2	3.45	2	4.65	0.76	0.87
ESS total score^a^	12.00 (3.00; 22.00)		8.00 (0.00; 23.00)		0.01	0.03
ISI total score^a^	18.00 (5.00; 27.00)		18.00 (4.00; 28.00)		0.76	0.53
BDI II total score^a^	12.00 (1.00; 45.00)		10.50 (1.00; 39.00)		0.42	0.74
Age at RLS symptom onset, years^a^	38.00 (6.00; 66.00)		47.50 (20.00; 71.00)		0.002	–
Age at daily RLS symptom onset, years^a^	45.00 (15.00; 73.00)		53.00 (30.00; 73.00)		0.02	–
RLS family history, Yes	17	32.08	21	53.85	0.04	0.04
RLS severity scale^a^	26.50 (15.00; 37.00)		26.50 (15.00; 40.00)		0.82	0.97
RLS severity scale						
11–20	5	8.93	9	21.43	0.05	0.05
21–30	36	64.29	17	40.48		
> 30	15	26.79	16	38.10		
**Polysomnography measurements**						
Total sleep time, min^a^	348.00 (119.00; 480.00)		298.00 (53.00; 443.00)		0.003	0.048
Sleep efficiency, %^a^	76.55 (24.94; 92.84)		64.10 (11.00; 6,300.00)		0.47	0.54
Wake time after sleep onset, min^a^	84.00 (15.00; 353.00)		132.00 (13.00; 404.00)		0.002	0.048
Sleep latency, min^a^	15.00 (1.00; 130.00)		20.00 (0.00; 300.00)		0.32	0.34
REM sleep latency, min^a^	82.00 (12.00; 323.00)		116.00 (39.00; 453.00)		0.04	0.11
N1, %^a^	7.00 (2.00; 29.70)		7.00 (1.88; 25.34)		0.32	0.80
N2, %^a^	54.84 (30.69; 77.93)		54.78 (32.70; 81.38)		0.98	0.67
N3, %^a^	17.98 (1.20; 35.03)		16.71 (0.00; 45.40)		0.67	0.38
REM sleep, %^a^	20.50 (0.40; 32.56)		17.90 (0.00; 43.21)		0.22	0.53
Microarousal index^a^	20.00 (3.00; 181.00)		31.00 (0.00; 170.00)		0.19	0.86
PLM during sleep index, /h^a^	35.48 (0.00; 234.32)		42.30 (0.00; 212.28)		0.55	0.62
PLM during sleep index, /h, ≥ 15	36	61.02	31	72.09	0.25	0.88
PLM during wake time index, /h^a^	29.71 (0.00; 161.19)		47.00 (0.00; 250.00)		0.01	0.11
Apnea–hypopnea index, /hour^a^	5.00 (0.00; 37.52)		7.85 (0.00; 55.84)		0.02	0.21
Apnea–hypopnea index, /h, ≥ 15	8	13.56	14	32.56	0.02	0.17
SaO_2_^a^	95.00 (91.00; 98.00)		95.00 (91.00; 97.00)		0.06	0.72
SaO_2_ less than 90% duration^a^	0.00 (0.00; 349.00)		0.00 (0.00; 32.00)		0.56	0.51
**Iron quantification**						
Serum ferritin, ng/mL^a^	113.00 (7.00; 500.00)		226.00 (26.00; 536.00)		< 0.0001	0.0001

*BMI* body mass index (weight in kg divided by the square of the body height in meters), *BDI* Beck Depression Inventory, *ESS* Epworth Severity Scale, *ISI* Insomnia Severity Index, *PLM* periodic legs movement, *REM* rapid eye movement, *SaO*_*2*_ oxygen saturation.

^a^Quantitative variables are expressed as medians (minimum value; maximum value).

Model 0: crude association, Model 1: adjustment for age.

### Serum hepcidin levels in patients with history of augmentation and in patients before and during treatment

In the RLS group, 17 patients (16.66%, 11 females) reported augmentation syndrome during the past 2 years with a median [range] of 82.12 days [14–464] before their participation in this study. However due to management of augmentation in out-patient clinic, none of them had augmentation at the time of study requiring a PSG recording in stable condition. Serum ferritin and hepcidin levels were not different between patients with (n = 17) and without (n = 85) recent history of augmentation (median [ranges] 180 [34–500] vs 147 [7–536]; 17.1 [4.8–39.6] vs 18.8 [0–77.2] respectively).

Thirty-four patients with RLS were evaluated again after a median interval of 13.78 months [0.88–25.52] during treatment with dopaminergic agonists (n = 29 with rotigotine alone; n = 2 with rotigotine and pregabalin; and n = 3 with pramipexole). After drug intake, the RLS severity, ESS, ISI and BDI scores improved as well as the PSG characteristics, including sleep efficiency, WASO, PLMS and PLMW (Table 4). Conversely, serum ferritin and hepcidin levels did not change after starting treatment. None of these 34 patients had augmentation at time of the second evaluation.

**Table 4:** Comparison of demographic, clinical, polysomnographic and iron measurements in patients with restless legs syndrome (RLS) before and during treatment.

Variable	Drug-free		Treated		p-value
	N = 34		N = 34		
	n	%	n	%	
**Demographic characteristics**					
Sex					
Male	15	44.12			
Female	19	55.88			
Age, years^a^	63.00 (34.78; 74.95)		63.09 (35.94; 76.25)		
BMI, kg/m^2^	26.59 (20.08; 30.52)		26.57 (20.44; 30.96)		0.29
ESS total score^a^	11.00 (3.00; 22.00)		8.50 (0.00; 23.00)		0.04
ISI total score^a^	17.00 (5.00; 27.00)		13.00 (1.00; 22.00)		< 0.0001
BDI II total score^a^	10.00 (2.00; 38.00)		7.00 (0.00; 35.00)		0.0008
RLS severity scale^a^	27.00 (18.00; 37.00)		16.00 (0.00; 29.00)		< 0.0001
RLS severity scale					
< 11	0	0.00	8	23.53	0.25
11–20	4	11.76	17	50.00	
21–30	18	52.94	9	26.47	
> 30	12	35.29	0	0.00	
**Polysomnography measurements**					
Total sleep time, min^a^	308.50 (53.00; 479.00)		365.50 (229.00; 453.00)		0.009
Sleep efficiency, %^a^	68.75 (11.00; 6,300.00)		76.49 (45.59; 96.33)		0.01
Wake time after sleep onset, min^a^	124.00 (31.00; 353.00)		83.00 (10.00; 204.00)		0.0001
Sleep latency, min^a^	12.50 (0.00; 63.00)		12.00 (0.00; 35.00)		0.25
REM sleep latency, min^a^	124.00 (12.00; 323.00)		100.00 (12.00; 248.00)		0.05
N1, %^a^	7.00 (3.78; 29.70)		7.21 (1.49; 19.22)		0.68
N2, %^a^	55.38 (30.69; 79.23)		62.04 (29.18; 77.93)		0.19
N3, %^a^	17.75 (0.95; 43.00)		14.06 (0.84; 39.73)		0.27
REM sleep, %^a^	18.42 (0.00; 29.92)		16.03 (5.41; 31.59)		0.42
Microarousal index^a^	40.00 (0.00; 181.00)		19.50 (5.00; 64.00)		< 0.0001
PLM during sleep index, /h^a^	61.45 (1.54; 234.32)		4.04 (0.00; 214.12)		< 0.0001
< 15	4	11.76	20	58.82	0.0002
≥ 15	30	88.24	14	41.18	
PLM during wake index, /h^a^	42.91 (3.53; 250.00)		22.73 (0.00; 135.97)		0.01
Apnea–hypopnea index, /h^a^	6.74 (0.00; 30.05)		7.58 (0.00; 39.23)		0.27
< 15	29	85.29	26	76.47	0.32
≥ 15	5	14.71	8	23.53	
SaO_2_^a^	95.00 (91.00; 97.00)		95.00 (92.00; 98.00)		0.30
**Iron quantification**					
Serum ferritin, µg/L^a^	193.00 (55.00; 500.00)		165.00 (55.00; 437.00)		0.09
Serum hepcidin, µg/L^a^	20.45 (3.08; 44.24)		17.34 (0.00; 59.47)		0.20

*BMI* body mass index (weight in kg divided by the square of the body height in meters), *BDI* Beck Depression Inventory, *ESS* Epworth Severity Scale, *ISI* Insomnia Severity Index, *PLM* periodic legs movement, *REM* rapid eye movement, *SaO*_*2*_ oxygen saturation.

^a^Quantitative variables are expressed as medians (minimum value; maximum value).

### Comparison of methods of quantification of hepcidin

**Figure 1:**
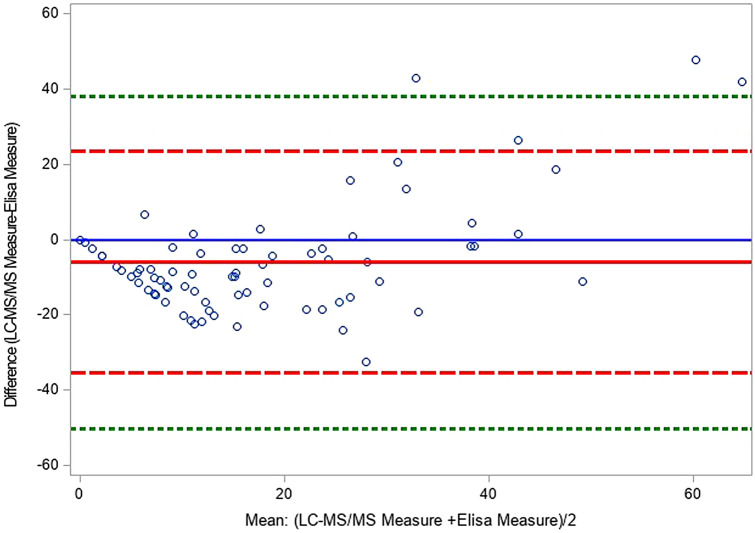
Bland–Altman plot of the difference (Measure_LC–MS/MS_–Measure_Elisa_) vs. the mean (Measure_LC–MS/MS_–Measure_Elisa_)/2 of the two methods of measurement for serum hepcidin. The mean of difference (solid red line), and dotted lines at ± 2 standard deviation (SD) which provide an estimate of where 95% of the difference should lie. Limits set at ± 3 SD (green dashed lines) provided an estimate for 99% of the differences and might provide a better outlier detector.

We quantified serum hepcidin levels using two different methods by LC–MS/MS assay as previously reported^9,26^ and by ELISA (the current method) in 42 patients with RLS (27 women, median age 63.00 years [34.78–77.22]) and 33 controls (19 women, median age 53.91 years [23.46–74.87]). The Bland–Altman plot showed that four individuals were out of the agreement limits (Fig. 1). Hepcidin level was lower when measured with the LC–MS/MS assay than with the ELISA method (mean difference = − 6.05 95% CI [− 9.44; − 2.66] p = 0.0007). Concerning the intra-individual reproducibility, a significant difference in the variability between the two methods was found (Pitman’s test of difference in variance, r = 0.552, p < 0.0001).

## Discussion

This study using a validated ELISA assay showed that serum hepcidin levels are higher in patients with RLS compared with healthy controls, in both unadjusted and adjusted models, and also after excluding subjects with low ferritin levels. This higher serum hepcidin level was not associated with dopaminergic drug intake and with recent history of augmentation. Conversely, serum ferritin levels did not differ between patients with RLS and controls, neither after dopaminergic drug intake nor augmentation.

The 25-amino acid-long hepatic bioactive form of hepcidin is released after proteolytic cleavage of the 84-amino acid-long prohepcidin precursor and plays a major role in iron homeostasis. Hepcidin-25 synthesis is inhibited by erythropoietic demand, hypoxia, and low iron content, while inflammation and high iron content stimulate its expression^17,27^. Hepcidin-25 decrease mediates the internalization and subsequent degradation of the iron cell exporter ferroportin, leading to cellular iron retention. Although detectable in biological fluids, the role of the other hepcidin isoforms (20, 22, 24 amino acids in length) remains unknown.

In the last few years, different immunochemical and mass spectroscopy-based assays have been used to quantify serum hepcidin-25 level in humans, but its clinical relevance remains to be determined. We previously found using LC–MS/MS in the MRM mode (the reference method for peptide quantification^9,26^) that serum hepcidin levels were higher in patients with RLS than in controls, and were correlated with RLS severity (non-linear relationship), and with PLMS. By using an ELISA assay with a monoclonal antibody, another reliable but less expensive method for hepcidin quantification that could be easily implemented in clinical settings^28^, we confirmed the higher serum hepcidin levels in patients with RLS than in controls. These results remained significant even after exclusion of participants included in the previous hepcidin measurement RLS study tested by LC–MS/MS study^9^. Here, the population of consecutive patients with RLS was diverse, in terms of severity, range of ferritin levels, dopaminergic drug intake, and recent history of augmentation syndrome. The between-group difference remained significant even after adjustment for confounding factors, and after excluding participants with ferritin levels below 50 µg/L. Indeed, participants with low ferritin levels were not excluded, but those taking ongoing iron replacement therapy or in the last 2 years.

Comparison of the two methods (LC–MS/MS and ELISA) showed that the ELISA-based test gave higher values, possibly due to immunoassay interference with the recognition of other hepcidin isoforms by the used antibody, in agreement with previous reports^29^. Despite the absence of standardized cut-offs for serum hepcidin levels, high serum hepcidin levels (*≥ *20.99 ng/mL) were associated with older age, older age at RLS onset, positive family history of RLS, lower excessive daytime sleepiness; however these results should be taken with caution regarding the multiple testing performed and the sample size of the population. The best hepcidin cut off value to discriminate patients with RLS from controls for hepcidin level was 18.1 µg/L (sensitivity of 52% and specificity of 75%) leading to a moderate satisfactory classification performance (AUC = 0.62 [0.55–0.71]).

Although serum ferritin is considered as the most informative RLS biomarker, in our study ferritin level was not different between patients with RLS and controls, as previously reported^9–13^. Moreover, the percentage of subjects with ferritin level < 50 ng/mL was similar in the RLS and control groups. However, identifying patients with low ferritin level remains important for RLS management, particularly because iron deficiency must be treated before considering treatment with a dopaminergic agonist. The value of measuring hepcidin before therapeutic management should be evaluated in future studies.

Serum ferritin and hepcidin levels were not different between patients tested before and during treatment with dopaminergic agonists, and between patients with and without recent history of augmentation syndrome. Conversely, a previous report showed an association between low ferritin levels and augmentation syndrome^14^. Our RLS group with augmentation syndrome was rather small, with retrospective diagnosis, and blood was not collected at the exact time of the augmentation syndrome, but after an interval of 82.12 days [range 14–464] due to management of augmentation in out-patient clinic (reducing or eliminating dopaminergic therapy, switching to alpha-2-delta ligands, opiates, or combined therapy), with study inclusion requiring PSG recording in stable condition. However, none of these 17 patients were treated with iron intake in the past two years. Serum hepcidin and ferritin levels do not change quickly from one month to another in the absence of associated specific conditions (e.g., iron intake, specific diet, inflammation, surgery) that did not apply to the patients included in the present study. Based on this limited data and its retrospective nature of assessment, further studies are required to elucidate whether hepcidin levels may predict subsequent development of augmentation.

Altogether, our results suggest that hepcidin may be a relevant RLS biomarker in terms of pathophysiology but less for diagnosis. The high serum hepcidin levels with normal ferritin concentrations in RLS may reflect a dysregulation of blood iron homeostasis rather than an absolute iron deficiency. This condition may mimic a functional peripheral iron deficiency, as we previously hypothesized^9^, thus limiting iron availability and its redistribution to different organs, including the brain, as part of RLS pathogenesis. However, hepcidin was assessed in serum samples, and may not reflect brain hepcidin concentrations^9^. High hepcidin levels have also been reported in other conditions (e.g., hemochromatosis^30^, metabolic syndrome^31^, atherosclerosis^32,33^, inflammation^34,35^, renal and cardiovascular diseases^36–38^) that may be associated with RLS, but almost not in our current RLS population due to our exclusion criteria. Indeed, recent studies strongly suggest that RLS should be seen as a continuous spectrum with a major genetic contribution, and a major environmental or comorbid disease contribution.

The present study has several strengths. We studied here consecutive patients with RLS, with diversity in terms of severity, range of ferritin levels, dopaminergic drug intake, and history of augmentation syndrome. All participants were well-characterized (RLS phenotype and comorbidities) and all underwent one-night PSG recording. We included patients with RLS associated with different conditions: idiopathic form, iron deficiency, drug-free and with treatment intake. A subgroup of patients with RLS was evaluated twice in similar condition (before and during treatment with dopaminergic agonists). Venous blood samples were collected between 7 and 8 a.m. after overnight fasting to avoid errors potentially related to circadian hepcidin variability. No participant had history of iron intake in the past 2 years. Hepcidin-25 was assessed using a reliable commercial ELISA assay that can be performed in the routine practice.

The present study also has some limitations. Despite some adjustments, unmeasured confounders, such as inflammatory and gastrointestinal conditions, could explain part of the associations observed with serum hepcidin level. Neither marker of inflammation, liver or renal function, no other measures of iron homeostasis were evaluated concomitantly with serum hepcidin and ferritin quantification. Seventy-five subjects (42 RLS patients and 33 controls) participated in a previous study that quantified serum hepcidin levels by LC–MS/MS, as comparison of methods of quantification of hepcidin was one secondary objective of the present study. Our clinical RLS population sample was well characterized but relatively small that may not reflective of RLS individuals from the general population. Finally, only a small subgroup of RLS patients was reassessed with a second clinical, biological and PSG evaluation under stable dopaminergic medication for RLS.

To conclude, using a reliable commercial ELISA assay, our study confirms higher serum hepcidin levels in patients with RLS, a biomarker that is not influenced by the treatment status or by recent history of augmentation. Due to the easy-to-use ELISA assay, hepcidin quantification could be implemented in the routine clinical practice, in view of a possible personalized approach before therapeutic management and potentially with hepcidin antagonists in some patients with RLS.

## Methods

### Population

One hundred and two drug-free consecutive adult patients with moderate to very severe RLS (63 women; median age 58.91 years [24.50–77.22]) were included. All patients underwent a face-to-face semi-structured evaluation and clinical examination by sleep experts to confirm RLS diagnosis and to exclude mimic conditions, using standard criteria as previously described^1,9^. Age at RLS onset, age at daily RLS onset, and family history of RLS were collected, as well as history of chronic diseases, such as hypertension, sleep apnea syndrome, cardiovascular, neurological, liver and renal diseases, diabetes and dyslipidemia.

None of patients had recent history (i.e. during the last 2 years) or ongoing iron intake, but iron deficiency (serum ferritin level < 50 ng/mL) discovered at time of study was not an exclusion criteria. All medications taken during the last month were recorded as well as their posology, and age at intake onset. No patient took drugs known to worsen (antidepressant, antihistaminic, or antipsychotic drugs) or alleviate (DAs, levodopa, alpha2-delta ligands, clonazepam and opioids) RLS symptoms for at least 2 weeks before study entry. All patients with RLS comorbid with clinically significant liver, renal, inflammatory, psychiatric or neurological diseases were excluded. RLS severity was evaluated according to the International Restless Legs Syndrome Study Group (IRLSSG) criteria (score ≤ 10: mild RLS; 11–20: moderate RLS; 21–30: severe RLS; and > 30: very severe RLS) as detailed elsewhere^9^. RLS augmentation during the last two years before the study was diagnosed retrospectively according to published criteria^16^.

Seventy-three community-dwelling adult controls (45 women, median age 56.85 years [23.46–76.65]) were recruited through advertisements and local association networks, during the same period, from the general population. All controls were free of RLS, liver, renal, inflammatory, psychiatric, and neurological diseases.

None of participants (patients and controls) took drugs known to affect RLS (dopaminergic agonists, L-DOPA, alpha-2-delta ligands, benzodiazepines or opioids), iron drugs, or any other medication known to influence sleep or movement (antidepressant, antihistaminic, or antipsychotic drugs) for at least 2 weeks before study inclusion as detailed elsewhere^9^.

All participants completed the Beck Depression Inventory II (BDI II)^21^ (score ≤ 13: minimal; 14–19: mild; 20–63: moderate-severe depression), the Epworth Sleepiness Scale (ESS)^22^ to evaluate daytime sleepiness (total score > 10: excessive daytime sleepiness), and the Insomnia Severity Index^23^ (ISI) (total score ≥ 15: moderate-severe insomnia).

This study was approved by Montpellier University review board, France, and all participants signed the informed consent. The study was performed in accordance with the Declaration of Helsinki and the French Good Clinical Practices.

### Polysomnography

All participants underwent a single one-night polysomnographic (PSG) recording in the sleep laboratory with six electroencephalogram leads, two electro-oculograms, one chin electromyogram, and electrocardiogram. Respiration was monitored with a nasal cannula/pressure transducer, mouth thermistor, chest and abdominal bands, and pulse oximeter. Sleep stages, microarousals and respiratory events were scored manually according to standard criteria as detailed elsewhere^9,24^. Surface electromyogram electrodes on the anterior tibialis muscles recorded limb movements (LM). PLMS and PLMW were scored according to the IRLSSG criteria, endorsed by the World Association of Sleep Medicine^25^.

A subgroup of 34 untreated patients with RLS (19 women, median age 63.0 years [34.8–74.9]) accepted on a voluntary basis to be reassessed in routine care with a second clinical, biological PSG evaluation under stable dopaminergic medication for RLS after a median interval of 13.78 months [0.88–25.52].

### Blood analyses

Venous blood samples were collected between 7:00 and 8:00 a.m. after overnight fasting, following standardized procedures. Serum ferritin levels were quantified within few hours after blood collection by electrochemiluminescence immunoassay (Elecsys, ECLIA, Cobas; Roche Diagnostics International Ltd, Switzerland). Serum samples from patients and controls were prepared in the same way and frozen at − 80 °C for hepcidin quantification. Hepcidin-25 (the 25-amino acid active isoform) was measured using the validated HS ELISA kit (DRG Diagnostics GmbH, Marburg, Germany). This assay relies on competitive binding detection, and was performed according to the providers' instructions (DRG, EIA-5782).

Hepcidin-25 was also measured by LC–MS/MS, as previously described, in a subgroup of 75 subjects (42 patients with RLS and 33 controls)^9,26^.

### Statistical analysis

Categorical variables are presented as numbers with percentages, and quantitative variables as medians with ranges. The Shapiro–Wilk test was used to test the normality of continuous variables. Regression logistic models were performed to compare demographic, clinical, and PSG characteristics between groups (controls and patients with RLS). Demographic variables significantly different in univariate analyses (p < 0.10) were included in logistic models to analyze the adjusted relationships between serum ferritin and hepcidin levels and the two groups. Associations were quantified with odds ratio (OR) and the 95% confidence interval (CI). The relationships of demographic, clinical and polysomnographic variables with hepcidin levels, categorized in two groups (≥ 20.99 µg/L that corresponds to the highest tertile in the RLS group versus < 20.99), were analyzed using logistic regression models. Hepcidin level was also dichotomized between patients with RLS and controls to compare the diagnostic performance using receiver operating characteristic (ROC) curves. The best possible threshold was defined by the highest Youden Index [(specificity (sp) + sensitivity (se)) − 1]. The Wilcoxon signed rank test was used to analyze differences between repeated and continuous measures (before and after treatment). The Cohen’s Kappa coefficient and the Bowker’s test of symmetry were used to test the agreement of categorical variables. Spearman’s rank order correlations were used to determine associations between continuous variables. The agreement of the two methods to measure hepcidin (LC–MS/MS and ELISA assays) was described using Bland–Altman plots and the intra-individual reproducibility of the method was analyzed using Pitman’s test. The significance level was set at p < 0.05. Analyses were performed using SAS (version 9.4; SAS Inc., Cary, NC, USA).

## References

[1] Allen RP, Picchietti DL, Garcia-Borreguero D (2014). Restless legs syndrome/Willis–Ekbom disease diagnostic criteria: Updated International Restless Legs Syndrome Study Group (IRLSSG) consensus criteria-history, rationale, description, and significance. Sleep Med..

[2] Allen RP, Walters AS, Montplaisir J (2005). Restless legs syndrome prevalence and impact: Rest general population study. Arch. Intern. Med..

[3] Montplaisir J, Boucher S, Poirier G, Lavigne G, Lapierre O, Lespérance P (1997). Clinical, polysomnographic, and genetic characteristics of restless legs syndrome: A study of 133 patients diagnosed with new standard criteria. Mov. Disord..

[4] Dauvilliers Y, Winkelmann J (2013). Restless legs syndrome: Update on pathogenesis. Curr. Opin. Pulm. Med..

[5] Trenkwalder C, Allen R, Högl B (2018). Comorbidities, treatment, and pathophysiology in restless legs syndrome. Lancet Neurol..

[6] Schormair B, Zhao C, Bell S (2017). Identification of novel risk loci for restless legs syndrome in genome-wide association studies in individuals of European ancestry: A meta-analysis. Lancet Neurol..

[7] Connor JR, Patton SM, Oexle K, Allen RP (2017). Iron and restless legs syndrome: Treatment, genetics and pathophysiology. Sleep Med..

[8] Lipschitz DA, Cook JD, Finch CA (1974). A clinical evaluation of serum ferritin as an index of iron stores. N. Engl. J. Med..

[9] Dauvilliers Y, Chenini S, Vialaret J (2018). Association between serum hepcidin level and restless legs syndrome. Mov. Disord..

[10] Lammers N, Curry-Hyde A, Smith AJ (2019). Are serum ferritin and transferrin saturation risk markers for restless legs syndrome in young adults? Longitudinal and cross-sectional data from the Western Australian Pregnancy Cohort (Raine) Study. J. Sleep Res..

[11] Didriksen M, Rigas AS, Allen RP (2017). Prevalence of restless legs syndrome and associated factors in an otherwise healthy population: Results from the Danish Blood Donor Study. Sleep Med..

[12] Benediktsdottir B, Janson C, Lindberg E (2010). Prevalence of restless legs syndrome among adults in Iceland and Sweden: Lung function, comorbidity, ferritin, biomarkers and quality of life. Sleep Med..

[13] Berger K, von Eckardstein A, Trenkwalder C, Rothdach A, Junker R, Weiland SK (2002). Iron metabolism and the risk of Restless Legs Syndrome in an elderly general population—The MEMO-Study. J. Neurol..

[14] Frauscher B, Gschliesser V, Brandauer E (2009). The severity range of restless legs syndrome (RLS) and augmentation in a prospective patient cohort: Association with ferritin levels. Sleep Med..

[15] Leu-Semenescu S, Petiau C, Charley Monaca C, Dauvilliers Y (2018). French consensus: Augmentation syndrome in restless legs syndrome. Rev. Neurol. (Paris)..

[16] Garcia-Borreguero D, Kohnen R, Silber MH (2013). The long-term treatment of restless legs syndrome/Willis–Ekbom disease: Evidence-based guidelines and clinical consensus best practice guidance: A report from the International Restless Legs Syndrome Study Group. Sleep Med..

[17] Hentze MW, Muckenthaler MU, Galy B, Camaschella C (2010). Two to Tango: Regulation of mammalian iron metabolism. Cell.

[18] Kroot JJC, Tjalsma H, Fleming RE, Swinkels DW (2011). Hepcidin in human iron disorders: Diagnostic implications. Clin. Chem..

[19] van der Vorm LN, Hendriks JCM, Laarakkers CM (2016). Toward worldwide hepcidin assay harmonization: Identification of a commutable secondary reference material. Clin. Chem..

[20] Zipperer E, Post JG, Herkert M (2013). Serum hepcidin measured with an improved ELISA correlates with parameters of iron metabolism in patients with myelodysplastic syndrome. Ann. Hematol..

[21] Beck AT, Steer RA, Carbin MG (1988). Psychometric properties of the Beck Depression Inventory: Twenty-five years of evaluation. Clin. Psychol. Rev..

[22] Johns MW (1991). A new method for measuring daytime sleepiness: The Epworth Sleepiness Scale. Sleep.

[23] Bastien CH, Vallières A, Morin CM (2001). Validation of the Insomnia Severity Index as an outcome measure for insomnia research. Sleep Med..

[24] Iber, C., Ancoli-Israel, S., Chesson, A. L., Quan, S. F. The AASM manual for the scoring of sleep and associated events: Rules, terminology and technical specifications. *Westchest Ill Am. Acad. Sleep Med.* (2007).

[25] Zucconi M, Ferri R, Allen R (2006). The official World Association of Sleep Medicine (WASM) standards for recording and scoring periodic leg movements in sleep (PLMS) and wakefulness (PLMW) developed in collaboration with a task force from the International Restless Legs Syndrome Study Group (IRLSSG). Sleep Med..

[26] Delaby C, Vialaret J, Bros P (2014). Clinical measurement of Hepcidin-25 in human serum: Is quantitative mass spectrometry up to the job?. EuPA Open Proteomics..

[27] Galesloot TE, Vermeulen SH, Geurts-Moespot AJ (2011). Serum hepcidin: Reference ranges and biochemical correlates in the general population. Blood.

[28] Uelker B, Cushman I, Dudek T, Herkert DM, Geacintov DCE (2016). High sensitivity hepcidin-25 bioactive elisas: Manual and fully automated system for the quantification of hepcidin-25 in human serum and plasma. Blood.

[29] Dahlfors G, Stål P, Hansson EC (2015). Validation of a competitive ELISA assay for the quantification of human serum hepcidin. Scand. J. Clin. Lab. Investig..

[30] Girelli D, Nemeth E, Swinkels DW (2016). Hepcidin in the diagnosis of iron disorders. Blood.

[31] Martinelli N, Traglia M, Campostrini N (2012). Increased serum hepcidin levels in subjects with the metabolic syndrome: A population study. PLoS ONE.

[32] Sullivan JL (2007). Macrophage iron, hepcidin, and atherosclerotic plaque stability. Exp. Biol. Med..

[33] Galesloot TE, Janss LL, Burgess S (2015). Iron and hepcidin as risk factors in atherosclerosis: What do the genes say?. BMC Genet..

[34] Nemeth E, Rivera S, Gabayan V (2004). IL-6 mediates hypoferremia of inflammation by inducing the synthesis of the iron regulatory hormone hepcidin. J. Clin. Investig..

[35] Nicolas G, Chauvet C, Viatte L (2002). The gene encoding the iron regulatory peptide hepcidin is regulated by anemia, hypoxia, and inflammation. J. Clin. Investig..

[36] Wagner M, Ashby D (2013). Hepcidin—a well-known iron biomarker with prognostic implications in chronic kidney disease. Nephrol. Dial. Transplant..

[37] van der Weerd NC, Grooteman MPC, Bots ML (2013). Hepcidin-25 is related to cardiovascular events in chronic haemodialysis patients. Nephrol. Dial. Transplant. Off. Publ. Eur. Dial. Transpl. Assoc. Eur. Ren. Assoc..

[38] Grammer TB, Scharnagl H, Dressel A (2019). Iron metabolism, hepcidin, and mortality (the Ludwigshafen Risk and Cardiovascular Health Study). Clin. Chem..

